# Supplementary material for: Imprinted Photonic Crystal-Film-Based Smartphone-Compatible Label-Free Optical Sensor for SARS-CoV-2 Testing
Source: Biosensors (Basel). 2022 Mar 28;12(4):200. doi: 10.3390/bios12040200 (PMC9026776; doi:10.3390/bios12040200)
Supplement: Supplementary file 1 [file biosensors-12-00200-s001.zip › biosensors-1658033-supplementary.pdf]

Supplementary Materials

Imprinted Photonic Crystal-Film-Based Smartphone-Compatible Label-Free Optical Sensor for SARS-CoV-2 Testing

Daiki Kawasaki [1], Hirotaka Yamada [1], Kenji Sueyoshi [1,2], Hideaki Hisamoto [1] and Tatsuro Endo [1,*]

[1] Department of Applied Chemistry, Graduate School of Engineering, Osaka Prefecture University, Sakai 599-8531, Japan; syb02029@edu.osakafu-u.ac.jp (D.K.); sxb02146@edu.osakafu-u.ac.jp (H.Y.); sueyoshi@chem.osakafu-u.ac.jp (K.S.); hisamoto@chem.osakafu-u.ac.jp (H.H.)

[2] Japan Science and Technology Agency (JST), Precursory Research for Embryonic Science and Technology (PRESTO), 5-3 Yonban-cho, Chiyoda, Tokyo 102-8666, Japan

* Correspondence: endo@chem.osakafu-u.ac.jp

Text S1. Functionalization of the IPCF surface

The anti-SARS-CoV-2 spike protein antibodies were chemically immobilized on the IPCF surface using the well-known modification process shown in Figure S1a.[34] First, the bare IPCF was air-plasma treated for 5 min. It was then salinized by APTES (1 wt% in 95% ethanol) to modify the surface with amino groups. Next, the surface was modified by GA (1 wt% in water) to produce aldehyde groups on the surface, followed by immobilization of the antibodies (1 ng/mL in PBS). Finally, the aldehyde groups on the surface were chemically blocked by EA (10 mM in PBS). SEM images of the bare, plasma-treated, and APTES-GA-coated IPCF surfaces are shown in Figure S1b. The hole diameter (N = 5) was increased from 231.0 nm to 304.0 nm by plasma treatment due to etching of the polymer by the air-plasma (100 W, 3 min), and then it was decreased to 284.0 nm by APTES-GA coating on the surface.

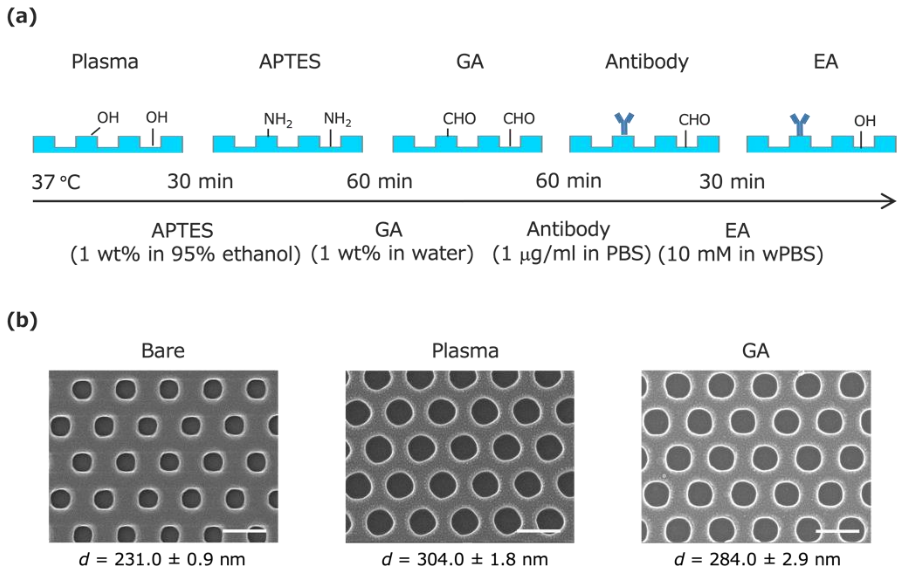

Figure S1. Functionalization of the IPCF surface. (**a**) Schematic illustration of the functionalization process. Only functional groups are described on the IPCF surface after each modification. (**b**) SEM images of the bare, plasma-treated, and APTES-GA coated IPCF surfaces. The diameter of the holes is described below each image (N = 5). The scale bar represents 500 nm.

Text S2. Evaluation of relative standard deviation values in the quantification of spike proteins

The RSD values for the quantification of spike proteins (Figure 5e, f) were evaluated. The RSD was determined as

$$RSD = \frac{SD}{\Delta R}, \qquad (eq.S1)$$

where SD is the standard deviation of the response ΔR. The RSD values for PBS and A. saliva are shown in Figure S2. The RSD values in the case of PBS were higher than those in the case of A. saliva at every concentration of spike proteins. This can be attributed to the effect of contaminants in A. saliva.

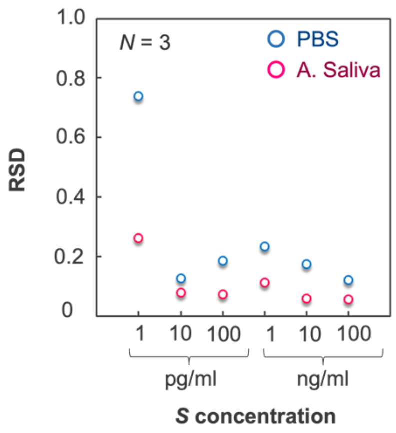

Figure S2. Relative standard deviation values in the quantification of the spike proteins in the case of PBS (Blue circle) and A. saliva (Magenta circle).

Text S3. Theoretical evaluation of responsivity.

The response of decreased diffracted light is attributed to the increase in the scattering on the diffracting surface, that is, the IPCF surface, due to the adsorbed molecules. Thus, if the dielectric characteristics are equal, the size of the adsorbate has a critical effect on the response. To investigate the response to the dielectric matter adsorbed onto the IPCF surface, the responsivity to the size of the dielectric particles on the surface was investigated by theoretical calculations based on the FDTD method. Figure S3a shows the surface (x-y) view of the calculation model of the IPCF (n = 1.5) with dielectric particles (n = 1.5, diameter = ds) set at an arbitrary position on the surface. The incident angle and polarization were 45°. The simulated reflection spectrum of the IPCF model was compared with the experimental spectrum (Figure S3b). The differences in the peak wavelength and spectral width were estimated to be caused by the detection angle and structural difference, respectively. It was confirmed that the polarization and incident angles did not shift the peak wavelength. Then, the responsivity of the dielectric particles was investigated. Here, the responses to a particle with a variety of diameters, ds, were calculated to investigate the effect of the adsorbate size on the responsivity of the IPCF sensor. Figure S3c shows the electric field distributions of the IPCF surface with and without particles on the surface at the peak wavelength. As the diameter of the particles increased, the scattering intensity around the particles increased. The responsivity to each particle diameter in the range of 10–200 nm is plotted in Figure S3d. In the case where a particle was in a hole, the responsivity was much less than that in the case on the surface. Next, we discuss the responsivity of the particles on the surface. In the diameter range from 0 to 100 nm, the responsivity exponentially increased, while in the diameter range of 100 to 200 nm, it linearly increased. It was expected that scattering by the particles critically degrades the diffraction when the particle size is above the diffraction limit. The responsivity of a particle with a diameter of 100 nm was approximately 200 times higher than that of a particle with a diameter of 20 nm. The theoretical responsivity to the particles adsorbed on the surface was investigated, and we discuss the difference between a SARS-CoV-2 particle and its spike proteins. It is known that the spike proteins form a trimer of size approximately 20 nm, while a SARS-CoV-2 particle has 20–30 spike proteins on its surface, and the particle size is approximately 100 nm.[36] In terms of the sizes of viruses and spike proteins, it is expected that the responsivity of the IPCF sensor to viruses is two orders of

magnitude higher than that to spike proteins. In terms of the number of viruses and spike proteins, 5,000 molecules of spike proteins, which were converted to molecular amounts from the LOD value, was equal to approximately 200 virus particles. Although these comparisons in terms of size and amount do not consider other factors that affect the responsivity of the IPCF sensor, such as the particle density, dielectric character, and aggregation of the targets, it is expected that the IPCF sensor has high sensitivity to SARS-CoV-2 as well as spike proteins. Some aspects of the simulation analysis should be discussed:

1. The adsorbate on the surface was a constant dielectric particle, while viruses and spike proteins have different dielectric characteristics and conformations.
2. The particles were periodically arrayed along the x-y direction at a constant position in the lattice, while the target particles were randomly adsorbed on the surface in the experiments. These two factors would decrease the responsivity compared to the experimental results.
3. The incident or polarization angle and particle position affect the responsivity quantitatively but not qualitatively. Therefore, theoretical investigation qualitatively estimates the sensitivity to spike proteins and viruses.

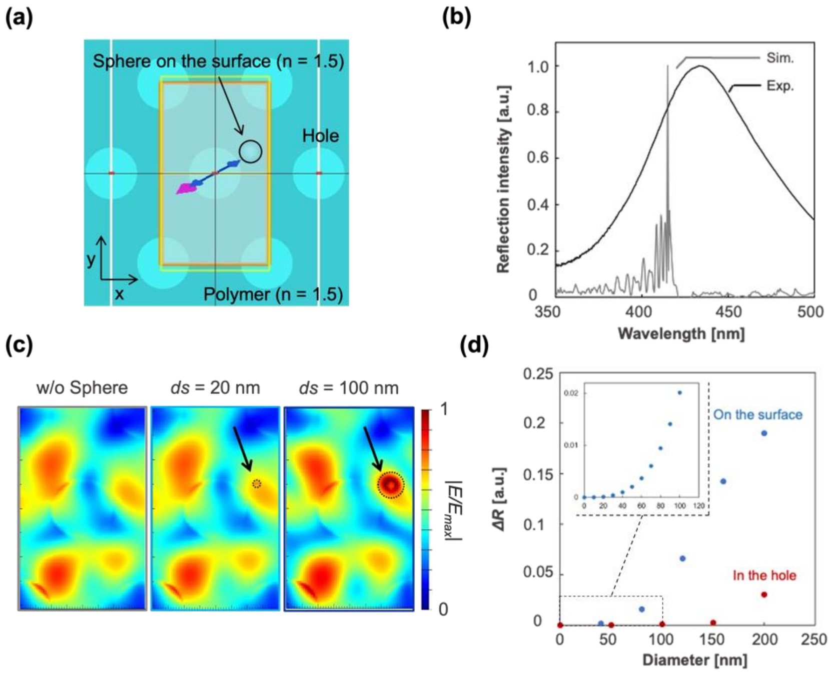

Figure S3. Theoretical evaluation of the responsivity to particles adsorbed on the surface. (a) Graphical surface of the simulation model of the IPCF, which consists of the polymer membrane (n = 1.5) and periodic air holes (diameter: 230 nm, lattice constant: 460 nm, and depth: 200 nm). The dielectric particle (n = 1.5) with diameter *ds* was positioned on the surface at the black circle. (b) Simulated (Grey line) and expeimental (Black line, extracted from the data in **Figure** 4a) reflection spectra of the IPCF. (c) Electric field distributions of the surface without and with the particle (*ds* = 20 and 100 nm) at the peak wavelength. The black arrow indicates the position of the particle. The intensity distribution is displayed by the factor $|E/E_{max}|$, where E and E_{max} are the electric field and its maximum, respectively. (d) Response to the particle on the surface (Blue dots) and in a hole (Red dots) with the variety of the diameters. The inset shows the detailed results in the diameter range from 0 to 100 nm.
